# Inhibition of EP2/EP4 signaling abrogates IGF-1R-mediated cancer cell growth: Involvement of protein kinase C-θ activation

**DOI:** 10.18632/oncotarget.3104

**Published:** 2014-12-31

**Authors:** Tetsuyuki Takahashi, Hisanori Uehara, Hirohisa Ogawa, Hitomi Umemoto, Yoshimi Bando, Keisuke Izumi

**Affiliations:** ^1^ Department of Molecular and Environmental Pathology, Institute of Health Biosciences, University of Tokushima Graduate School, Tokushima, Japan; ^2^ Division of Pathology, Tokushima University Hospital, Tokushima, Japan

**Keywords:** E-prostanoid receptor, insulin-like growth factor receptor-1, protein kinase C-θ

## Abstract

Associations between growth factor receptor-mediated cell signaling and cancer cell growth have been previously characterized. Receptors for prostaglandin E_2_, such as EP2, and EP4, play roles in cancer growth, progression and invasion. Thus, we examined the interactions between EP2/EP4- and IGF-1R-mediated cellular signaling in human pancreatic cancer cells. Selective antagonists against EP2 and EP4 abrogated IGF-1-stimulated cell growth and suppressed MEK/ERK phosphorylation. In subsequent experiments, phospho-antibody arrays indicated increased phosphorylation levels of protein kinase C-θ (PKC-θ) at the Thr538 position following the inhibition of EP2/EP4-mediated signaling. Inhibition of PKC-θ activity impaired cell viability compared with EP2/EP4-antagonized IGF-1-stimulated cells. PKC-θ kinase MAP4K3, which plays a pivotal role in PKC-θ activation, also affected growth signaling in the presence of EP2/EP4 antagonists. Administration of EP2 and EP4 antagonists significantly inhibited the growth of an orthotopic xenograft of IGF-1-secreting pancreatic cancer cells, with increased phospho-PKC-θ and decreased phospho-ERK. Clinico-pathological analyses showed that 17.4% of surgical pancreatic cancer specimens were quadruple-positive for IGF-1R, EP2 (or EP4), MAP4K3, and PKC-θ. These results indicate a novel signaling crosstalk between EP2/EP4 and IGF-1R in cancer cells, and suggest that the MAP4K3-PKC-θ axis is central and could be exploited as a molecular target for cancer therapy.

## INTRODUCTION

Growth factor receptor-mediated signaling is known to be important for cancer cell growth, tumor development, metastasis and various other events in several types of cancer, including pancreatic carcinomas [1-5]. Therefore, most therapeutic molecular targets involve the kinase activities of growth factor receptors and their ligands [6]. Insulin-like growth factor-1 and -2 (IGF-1 and -2, respectively) and their receptors (IGF-1R) are potential targets that trigger multiple intracellular signaling pathways [5, 7]. The circulating IGF-1 levels are closely associated with the risk of cancer [8, 9], and the blockade of IGF-1R, using anti-IGF-R1 antibodies, IGF1R antisense, and inhibitory analogues of IGF-1 suppress tumor cell growth and angiogenesis [10-13]. In patients with several cancers including pancreatic cancer, high expression of IGF-1R in tumors is associated with higher tumor grades and poor survival [14, 15]. *In vitro* studies have shown that exogenous IGF-1-stimulated growth of pancreatic cancer cell lines is abrogated following treatment with anti-IGF1R antibodies [16].

Prostaglandins such as prostaglandin E_2_ (PGE_2_), are also associated with cancer cell growth, tumor development and metastasis, as well as with inflammation and other physiological events [17,18]. Cycooxygenase-2 (COX-2) is an inducible enzyme that converts arachidonic acid into prostaglandins, and its roles in the development of many tumor types have been demonstrated in genetic and inhibitor studies, histopathological analyses, and epidemiological studies [19-23]. PGE_2_ receptors or E-prostanoid receptors (EPs) comprise several subtypes (EP1-EP4), which can be classified into three types based on their signaling features [24]. Both basic and clinical studies have reported increased PGE_2_ production and the overexpression of EPs in tumor tissues in pancreatic cancer, as well as in a wide range of cancers [25, 26]. Therefore, EP-mediated cellular signaling may be a potent antitumor target, which could be exploited using specific antagonists of EPs or COX.

Numerous interactions between growth factor receptor-mediated signaling pathways have been shown to play pivotal roles in accelerated cancer cell growth, invasion, and metastasis. In particular, the interactions between IGF-1R, epidermal growth factor receptor (EGFR), platelet-derived growth factor receptor, and estrogen receptors have been reported to synergistically potentiate cell proliferation [27-29]. Moreover, the transactivation of EP and EGFR, and the subsequent activation of mitogenic signaling have also been demonstrated in several cancers [30-32]. However, reciprocal combinations between EPs and other growth factor receptors, including IGF-1R, have not been fully elucidated. In the present study, we checked for the presence of alternative signaling interactions between EPs and IGF-1R mainly in pancreatic cancer cells using selective antagonists against EP2 and EP4. Thereafter, phospho-antibody arrays were used to determine the molecular relationships between EPs and IGF-1R signaling, where *in vivo* experiments and clinico-pathological analyses were performed to demonstrate the molecular basis and probability of this signaling crosstalk.

## RESULTS

### Characteristics of human pancreatic cancer cells

Initially, the expression of 12 parameters was examined in the pancreatic cancer cell lines MiaPaCa-2, BxPC-3, PANC-1, and Capan-1 by RT-PCR, and the secretions of PGE_2_ in culture media (CM) were determined using EIA. COX-1 and COX-2 mRNA expression was observed in BxPC-3 cells.

**Figure 1:**
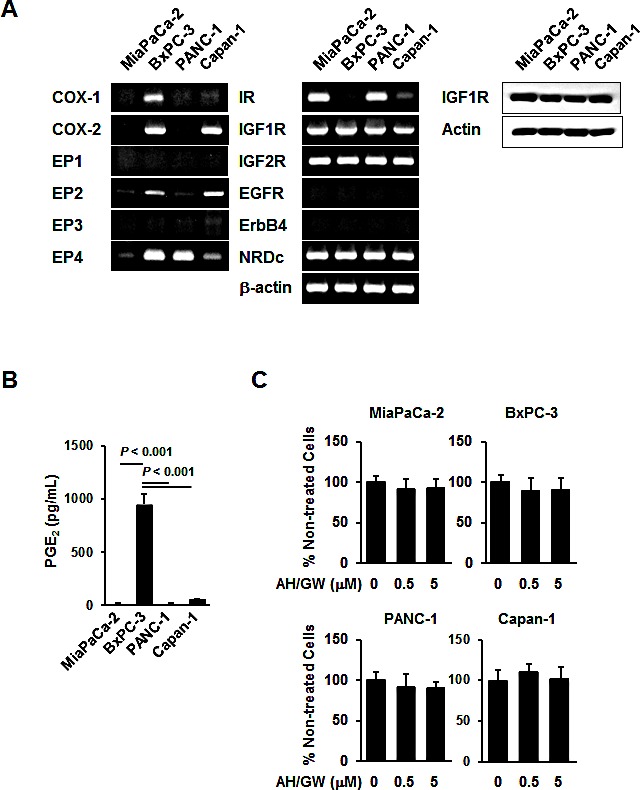
Expression patterns and responses to EP2/EP4 antagonists AH6809/GW627368X in pancreatic cancer cell lines A, The levels of COX-1, COX-2, EP1-EP4, IR, IGF-1R, IGF-2R, EGFR, ErbB4, NRDc, and β-actin mRNA and IGF-1R protein were measured in MiaPaCa-2, BxPC-3, PANC-1, and Capan-1 human pancreatic cancer cell lines. B, CM (serum-free for 48 h) from these cell lines were also subjected to PGE_2_ enzyme immunoassays. *Columns*, mean values (*n* = 4) based on two independent experiments; *bars*, SD. C, Pancreatic cancer cell lines were treated with 0.5 and 5 μM AH6809/GW627368X for 48 h and the cell viabilities were measured using the MTT assay. The A_550_ values for untreated cells were assigned as 100% and the relative percentages for treated cells are shown. *Columns*, mean percentages (n = 6); *bars*, SD.

Capan-1 cells expressed COX-2 mRNA, whereas MiaPaCa-2 and PANC-1 cells did not. Therefore, EIA analyses revealed the highest PGE_2_ levels in BxPC-3 CM (over 10 times than that in the other cell lines, Fig 1B). MiaPaCa-2 cells expressed EP4 mRNA at very low levels, whereas BxPC-3 cells exhibited low expression of EP2 mRNA and high expression of EP4 mRNA. Only high EP4 mRNA expression was observed in PANC-1 cells, whereas moderate EP2 mRNA expression and weak EP4 mRNA expression were observed in Capan-1 cells (Fig 1A). In subsequent experiments, similar levels of IGF-1R mRNA and protein, IGF-2R, and NRDc mRNA were expressed in all cell lines. However, IR mRNA expression was detected only in MiaPaCa-2 and PANC-1 cells, whereas EGFR and ErbB4 mRNAs were not expressed in any of the four cell lines (Fig 1A).

### Antagonism of EP2/EP4 signaling blocks IGF-1-induced growth stimulation in BxPC-3 cells

**Figure 2:**
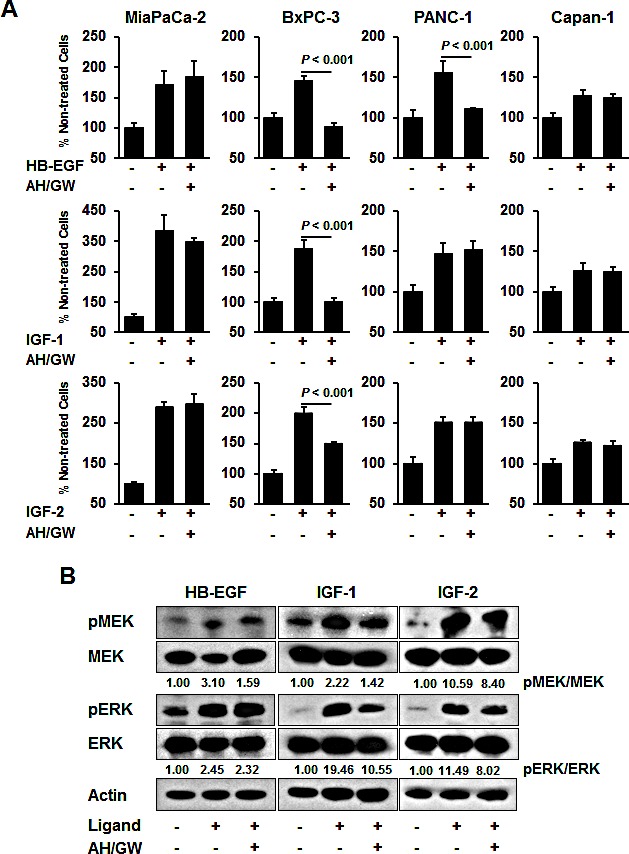
Effect of AH6809/GW627368X on HB-EGF-, IGF-1-, and IGF-2-stimulated growth stimulation in pancreatic cancer cell lines A, Cells were stimulated with HB-EGF (50 ng/mL), IGF-1 (20 ng/mL), or IGF-2 (50 ng/mL) for 48 h in the absence or presence of AH6809/GW627368X (5 μM each) pretreatment for 3 h and cell growth was measured using the MTT assay. The A_550_ values for untreated cells were assigned as 100% and the relative percentages for the treated cells are shown. *Columns*, mean percentages (*n* = 6); *bars*, SD. B, BxPC-3 cells were stimulated with HB-EGF (50 ng/mL), IGF-1 (20 ng/mL) and IGF-2 (50 ng/mL) for 20 min in the absence or presence of AH6809/GW627368X (5 μM each) pretreatment for 3 h. The levels of phosphorylated MEK, total MEK, phosphorylated ERK, and total ERK were determined by immunoblotting. The relative levels of phospho-MEK and -ERK were calculated using ImageJ software.

To investigate the effects of the antagonism of EP2/EP4-mediated cellular signaling on growth factor receptor-mediated growth stimulation, we treated cells with EP2/EP4 antagonists, i.e., AH6809/GW627368X, and assessed HB-EGF-, IGF-1-, and IGF-2-mediated growth stimulation. Single treatments with AH6809/GW627368 did not significantly affect cell viability in any of the cell lines (Fig 1C). Growth factor treatments increased the cell viability in all lines, but the degree of stimulation was relatively low in Capan-1. In BxPC-3 cells, pretreatment with AH6809/GW627368X completely blocked the growth stimulated by HB-EGF and IGF-1 and partially inhibited stimulation following treatment with IGF-2. In PANC-1 cells, pretreatment with AH6809/GW627368X only blocked HB-EGF-mediated growth stimulation, whereas growth stimulation by these growth factors was not affected by pretreatment with AH6809/GW627368X in MiaPaCa-2 and Capan-1 cells (Fig 2A). We also tested single treatments of AH6809 or GW627368X and assessed IGF-1-mediated growth stimulation in BxPC-3 cells. Pretreatment with GW627368X almost completely blocked growth stimulation by IGF-1 and pretreatment with AH6809 also suppressed it to a low but significant extent (Supplementary Fig 2A). Based on these results, we decided that it was appropriate to use AH6809/GW627368X in combination to achieve absolute inhibition of EP2/EP4-mediated cellular signaling. The same experiment was conducted using human breast cancer cell lines MCF-7 and MDA-MB-231, and human prostate cancer cell lines PC-3, DU145, and LNCaP. Similar inhibitory effects were also found in MCF-7 and DU145 cells, as well as in BxPC-3 cells (Supplementary Fig 2B).

In BxPC-3 cells, treatment with growth factors induced the phosphorylation of MEK and ERK, whereas pretreatment with AH6809/GW627368X attenuated their phosphorylation in IGF-1-treated cells (Fig 2B). Single treatments with AH6809/GW627368 did not affect the phosphorylation of MEK and ERK (Supplementary Fig 1). No changes were observed in HB-EGF-treated cells and phosphorylation decreased only marginally in IGF-2-treated cells (Fig 2B). EP4 knockdown also abrogated IGF-1-induced cell growth and the phosphorylation of MEK and ERK with similar efficacy to that following pretreatment with AH6809/GW627368X (Supplementary Fig 3A).

### Phosphorylation of protein kinase C-θ (PKC-θ) is induced by antagonism of EP2/EP4 signaling and it plays a role in cell growth and survival

**Figure 3:**
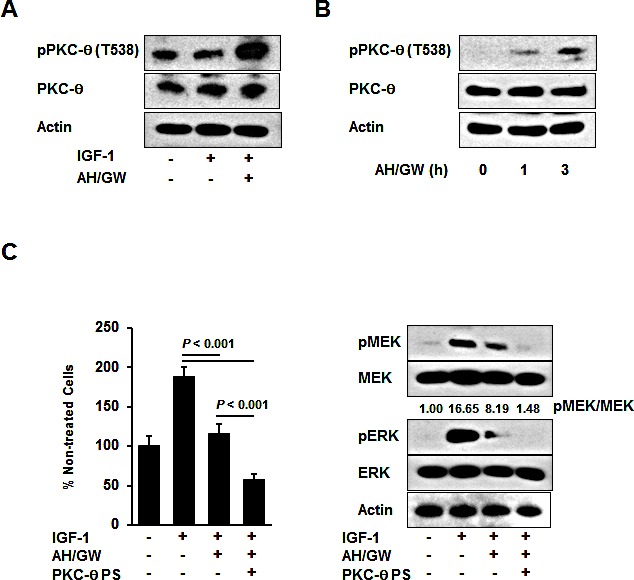
Involvement of PKC-θ phosphorylation in the antagonism of EP2/EP4 signaling A, BxPC-3 cells were stimulated with IGF-1 (20 ng/mL) for 20 min in the absence or presence of AH6809/GW627368X (5 μM each) pretreatment for 3 h. The levels of phosphorylated PKC-θ and total PKC-θ were determined by immunoblotting. B, BxPC-3 cells were treated with AH6809/GW627368X (5 μM each) for 1 and 3 h. The levels of phosphorylated PKC-θ and total PKC-θ were determined by immunoblot analysis. C, A pseudosubstrate of PKC-θ (10 μM, Millipore) was added with AH6809/GW627368X (5 μM each) pretreatment and the cell growth and phosphorylation of MEK and ERK were determined by MTT assays and immunoblotting, respectively. The A_550_ values for untreated cells were assigned as 100% and the relative percentages for treated cells are shown. The relative levels of phospho-MEK and -ERK were calculated using ImageJ software. *PS*, pseudosubstrate; *Columns*, mean percentages (*n* = 6); *bars*, SD.

**Figure 4:**
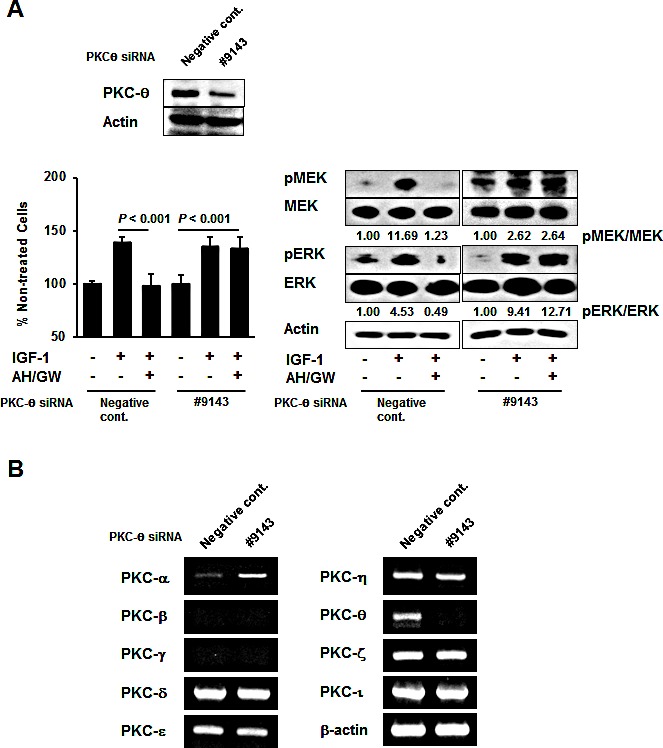
PKC-θ knockdown offsets the effects of the antagonism of EP2/EP4 signaling by compensatory induction of PKC-α A, Knockdown using PKC-θ siRNA was performed in BxPC-3 cells and confirmed by immunoblotting. Cells transfected with negative control siRNA and PKC-θ siRNA were stimulated with IGF-1 for 48 h or 20 min in the absence or presence of AH6809/GW627368X pretreatment for 3 h, and then tested by growth stimulation assays and immunoblotting. The A_550_ values for untreated cells were assigned as 100% and the relative percentages for treated cells are shown. The relative levels of phospho-MEK and -ERK were calculated using ImageJ software. *Columns*, mean percentages (n = 6); *bars*, SD. B, The expression levels of PKC-α, PKC-β, PKC-γ, PKC-δ, PKC-ε, PKC-η, PKC-θ, PKC-ζ, PKC-ι, and β-actin mRNA were determined in BxPC-3 cells transfected with negative control siRNA and PKC-θ siRNA.

Phospho-antibody arrays were used to assess changes in the protein phosphorylation status to identify molecules that are affected by antagonism of EP2/EP4 signaling in IGF-1-treated BxPC-3 cells. Compared with IGF-1-treated cells, three induced proteins and five inhibited proteins were identified in AH6809/GW627368X-pretreated IGF-1-treated cells with cut-off and fold changes of >2.0 and <0.5, respectively (Table 1). The protein that changed the most was PKC-θ, which exhibited an 8.628-fold increase in the phosphorylation of Thr538. Consistent with this observation, immunoblotting detected increased phospho-PKC-θ at Thr538 in AH6809/GW627368X-pretreated IGF-1-treated cells compared with IGF-1-treated cells and untreated cells (Fig 3A). Moreover, treatments with AH6809/GW627368X alone induced the phosphorylation of PKC-θ in a time-dependent manner (Fig 3B). EP4 knockdown increased the phosphorylation of PKC-θ with similar efficacy to that following AH6809/GW627368X pretreatment (Supplementary Fig 2A). Because the phosphorylation of PKC-θ at Thr538 is associated directly with kinase activity [33], we examined the effect of PKC-θ inhibition using a pseudo-substrate as a specific inhibitor on growth stimulation following the antagonism of EP2/EP4 signaling. Inhibition of the basal activity of PKC-θ by a pseudo-substrate suppressed IGF-1-mediated growth stimulation (data not shown). Together with the AH6809/GW627368X pretreatments in the presence of the pseudo-substrate, the number of IGF-1-treated viable cells further decreased by approximately 43% and the MEK and ERK phosphorylation levels also reduced (Fig 3C). However, according to both growth stimulation assays and immunoblotting, the knockdown of PKC-θ using a specific siRNA impaired the effect of AH6809/GW627368X pretreatment (Fig 4A). Finally, RT-PCR demonstrated the compensatory expression of PKC-α mRNA in PKC-α siRNA-transfected cells (Fig 4B).

**Table 1:** Results of phospho-antibody array (IGF-1R-relating molecules) in BxPC-3 treated with IGF-1 vs. with AH/GW+ IGF-1

molecule	Ratio (AH/GW+ IGF-1/IGF-1)
increased	
PKC-θ (Thr538)	8.6283
IKK-γ (Ser31)	2.9154
IRS-1 (Ser639)	2.0063
decreased	
Gab2 (Tyr643)	0.3796
IKK-α/β (Ser180/181)	0.4247
14-3-3 ζ/δ (Thr232)	0.4650
PKC-δ (Tyr52)	0.4825
PKC-δ (Tyr64)	0.4871

### MAP4K3 is involved in PKC-θ phosphorylation and the subsequent impairment of IGF-1-induced growth stimulation following the antagonism of EP2/EP4 signaling

**Figure 5:**
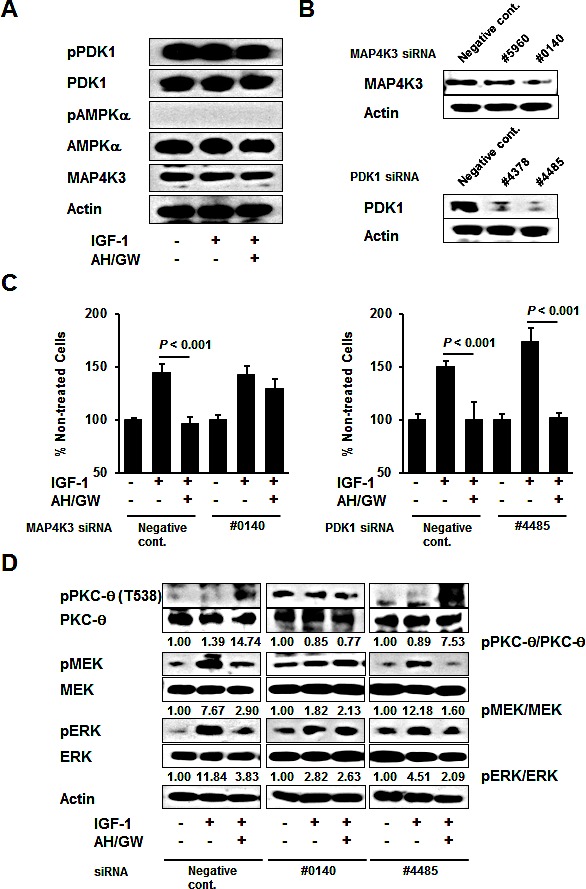
MAP4K3 plays a key role in the activation of PKC-θ by EP2/EP4 antagonism A, BxPC-3 cells were stimulated with IGF-1 (20 ng/mL) for 20 min in the absence or presence of AH6809/GW627368X (5 μM each) pretreatment for 3 h. The levels of phosphorylated PDK1, total PDK1, phosphorylated AMPKα, total AMPKα, and MAP4K3 were determined by immunoblotting. B, Knockdown was performed with specific siRNAs for MAP4K3 and PDK1 in BxPC-3 cells and confirmed by immunoblotting. C, Cells transfected with negative control siRNA, MAP4K3 siRNA (#0140), and PDK1 siRNA (#4485) were stimulated with IGF-1 (20 ng/mL) for 48 h in the absence or presence of AH6809/GW627368X (5 μM each) pretreatment for 3 h. Cell growth was measured by the MTT assay. The A_550_ values for untreated cells were assigned as 100% and the relative percentages for treated cells are shown. *Columns*, mean percentages (*n* = 6); *bars*, SD. D, Cells transfected with negative control siRNA, MAP4K3 siRNA (#0140), and PDK1 siRNA (#4485) were stimulated with IGF-1 (20 ng/mL) for 20 min in the absence or presence of AH6809/GW627368X (5 μM each) pretreatment for 3 h. The levels of phosphorylated PKC-θ, total PKC-θ, phosphorylated MEK, total MEK, phosphorylated ERK, and total ERK were determined by immunoblotting. The relative levels of phospho-PKC-θ, -MEK and -ERK were calculated using ImageJ software.

Further analyses of phospho-PDK1 and phospho-AMPKα were performed because the phosphorylation of PKC-θ is reportedly achieved by PDK1, AMPK, and MAP4K3 [34]. The phospho-PDK1 levels were not altered in IGF-1-treated cells or in AH6809/GW627368X-pretreated IGF-1-treated cells (Fig 5A). However, AMPKα phosphorylation was not induced by any of the treatments described above and the total protein levels of PDK1, AMPKα, and MAP4K3 were similar in the non-treated cells, IGF-1-treated cells, and AH6809/GW627368X-pretreated IGF-1-treated cells. Because commercial antibodies against phospho-MAP4K3 are currently unavailable, the MAP4K3 and PDK1 protein levels were knocked down using specific siRNAs (Fig 5B). MAP4K3 knockdown abrogated the suppressive effect of AH6809/GW627368X on IGF-1-stimulated cell growth compared with negative control siRNA- and PDK1 siRNA-transfected cells (Fig 5C). To support this observation, immunoblotting showed that MAP4K3 knockdown cells failed to phosphorylate PKC-θ or to suppress the phosphorylation of MEK and ERK following AH6809/GW627368X pretreatment and IGF-1 treatment (Fig 5D). Similar to that in AH6809/GW627368X-pretreated cells, the MAP4K3 knockdown abolished the suppression of IGF-1-stimulated cell growth, PKC-θ phosphorylation, and the inhibition of MEK and ERK phosphorylation in cells transfected with EP4 siRNA (Supplementary Fig 3B). In the cells transfected with negative control siRNA or PDK1 siRNA, pretreatment with AH6809/GW627368X increased the phosphorylation of PKC-θ and suppressed the phosphorylation of MEK and ERK (Fig 5D). In subsequent experiments, the specific PDK1 inhibitor BX912 had no effects on the AH6809/GW627368X-induced suppression of IGF-1-stimulated cell growth and PKC-θ, or MEK, and ERK phosphorylation (Supplementary Fig 4).

### Administration of AH6809/GW627368X suppresses IGF-1-secreting pancreatic tumor growth in an orthotopic nude mouse xenograft model

**Figure 6:**
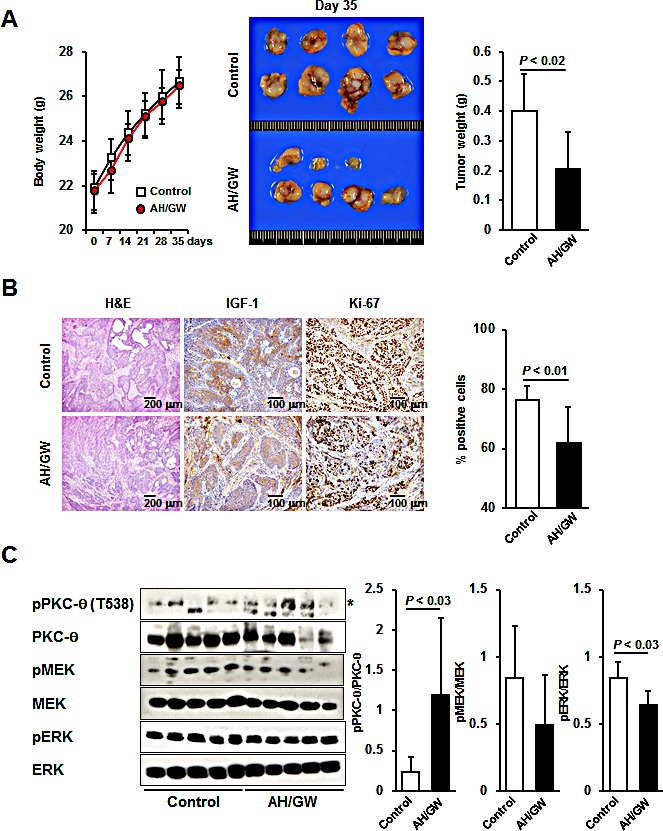
Effect of EP2/EP4 antagonism on IGF-1-expressing pancreatic tumor growth *in vivo* A, The body weights of mice were measured (left) at 0, 7, 14, 21, 28, and 35 days after injection. *Bars*, SD. Macroscopic tumors from control and AH6809/GW627368X-treated mice (center). The average tumor weights were calculated for each group (right). *Columns*, mean; *bars*, SD. B, H&E staining and immunohistochemical staining of IGF-1 and Ki-67 in tumor lesions. The percentage of Ki-67-positive cells was calculated. *Columns*, mean; *bars*, SD. C, Proteins from tumors were subjected to immunoblotting and the relative levels of phospho-PKC-θ, -MEK, and -ERK were calculated using ImageJ software. *Asterisk*, targeted band; *Columns*, mean; *bars*, SD.

We tested the effects of AH6809/GW627368X in an orthotopic nude mouse xenograft model to determine whether the aforementioned cellular events occured *in vivo* as well. The BxPC-3 cells did not express or secrete IGF-1 (data not shown), so we initially established stable transfectants expressing hmIGF-1 (BxPC-hmIGF-1). BxPC-hmIGF1 cells secreted hmIGF-1 into CM in an FBS-dependent manner. The growth rates of the BxPC-hmIGF1 transfectants were higher than those of the vector-control transfected cells (BxPC-mock), and treatments with AH6809/GW627368X decreased cell proliferation only in BxPC-hmIGF1 (Supplementary Fig 5A-C). Intrapancreatic injection of these cells caused tumor formation in both groups, with larger tumors in BxPC-hmIGF1-injected mice. The average tumor weights and serum IGF-1 levels in BxPC-hmIGF1-injected mice were significantly higher than those in BxPC-mock-injected mice (Supplementary Fig 5D). H&E staining and immunohistochemical staining for IGF-1 showed that these tumors were somewhat differentiated, and that IGF-1 expression was sustained during the experimental period. In addition, the number of Ki-67-positive cells (a marker for proliferative cells) significantly increased with the tumor weights in BxPC-hmIGF1-injected mice (Supplementary Fig 5E). Thus, the effects of AH6809/GW627368X on pancreatic tumor growth were examined in BxPC-hmIGF1-bearing mice. The mean body weights of AH6809/GW627368X-treated mice did not significantly change compared with control mice during the experimental period (Fig 6A). The incidence of the visible tumors did not change (80% in control mice and 70% in AH6809/GW627368X-treated mice); however, the tumor weights significantly decreased in AH6809/GW627368X-treated mice (Fig 6A). H&E and immunohistochemical staining for IGF-1 showed that treatment with AH6809/GW627368X did not alter the tumor types or IGF-1 expression (Fig 6B). Moreover, immunohistochemical staining for Ki-67 and the quantification of labeling indices indicated that the percentages of Ki-67-positive cells were significantly lower in AH6809/GW627368X-treated mice (Fig 6B). Next, the phosphorylation levels of PKC-θ, MEK, and ERK were examined by immunoblotting in tumor lesions. The relative expression levels of phospho-PKC-θ were significantly elevated in tumors treated with AH6809/GW627368X. The phospho-MEK levels decreased only marginally but a significant decrease in the phospho-ERK levels was observed (Fig 6C).

### Concomitant expression of IGF-1R, EP2/EP4, MAP4K3, and PKC-θ in specimens from pancreatic cancer patients

**Figure 7:**
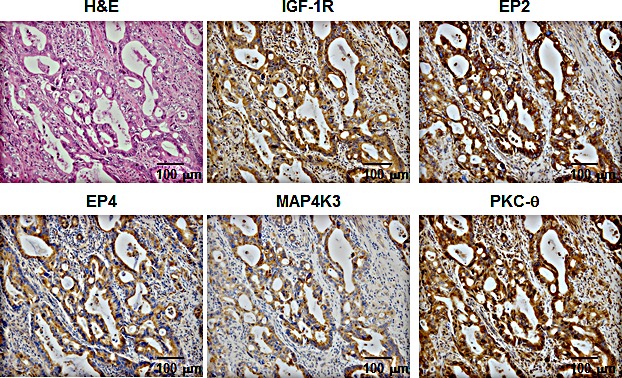
H&E and immunohistochemical staining for IGF-1R, EP2, EP4, MAP4K3, and PKC-θ in the representative case of quadruple positive The tumor specimen co-expressed all of the proteins.

To assess the physiological relevance of our *in vivo* observations, we examined the expression patterns of IGF-1R, EP2/EP4, MAP4K3, and PKC-θ in surgical specimens from pancreatic cancers based on immunohistochemical analyses. The pancreatic cancer specimens comprised two Grade I, one Grade II, 10 Grade III, and 10 Grade IV cancers. All of the specimens expressed EP2 and EP4, and 47.8%, 52.2%, and 60.9% of the specimens were positive for IGF-1R, MAP4K3, and PKC-θ, respectively (data not shown). Concomitant expression of all of these proteins was observed in 17.4% of the cancer specimens (data not shown). The representative case of quadruple positive is shown in Fig 7.

## DISCUSSION

Crosstalk between signaling pathways contributes to tumor progression, metastasis, and the acquisition of resistance to anti-cancer drugs. Thus, an improved understanding of these interactions may facilitate the identification of novel targets and the development of effective anti-cancer strategies. Although many patterns of signaling crosstalk have been demonstrated, only few signaling pathways have been associated with EPs. In the present study, we characterized a novel interaction between EP2/EP4 and IGF-1R signaling pathways mainly in pancreatic cancer cells.

Initially, we examined the mRNA expression patterns of COX, EP receptors, and several growth factor receptors in four pancreatic cancer cell lines. Among these cell lines, BxPC-3 cells exhibited the most marked COX- and EP4-dependent constitutive positive feedback loop and they secreted PGE_2_ into CM; thus, they were used in further experiments. Because IGF-1R, IGF-2R, and NRDc were expressed in BxPC-3 cells, we performed growth stimulation assays following treatment with IGF-1, IGF-2, and HB-EGF. Pretreatment with the EP2/EP4 antagonists AH6809/GW627368X completely blocked growth stimulation on treatment with IGF-1 and HB-EGF and partially blocked it following treatment with IGF-2. Similar reactions were observed in IGF-1-stimulated MCF-7 and DU145 cells, which expressed EP2, EP4, and IGF-1R, respectively (Supplementary Fig 2B and Ref. 35, 36). This result suggests that the similar interaction may occur not only in pancreatic cancer cells but also in various types of cancer cells including breast and prostate cancer, if both the feedback loop of COX-EP (2 and/or 4)-PGE_2_ production and IGF-1R expression exhibited. The partial suppression of IGF-2-stimulated cell growth may reflect as differences in the binding affinity of IGF-1R, which may offset the effects of IGF-2 by IGF-2R. In agreement with the growth stimulation assays, corresponding phosphorylation levels of MEK and ERK were confirmed. Moreover, the phosphorylation of Akt, which is a key molecule in the other main IGF-1-mediated signaling pathways, was not significantly altered under these conditions (data not shown). Overall, these data indicate the primary contribution of EP2/EP4 signaling to IGF-1R-mediated growth and MAPK signaling.

Kinase activation and subsequent phosphorylation are central components of cell signal transduction. The AH6809/GW627368X pretreatments blocked the phosphorylation and activation of MEK and ERK, so we performed phospho-antibody arrays that demonstrated the dramatic phosphorylation of PKC-θ Thr538, which presumably leads to increased kinase activity. PKC-θ plays essential roles in the regulation of peripheral T cell activation, prevention of T cell anergy, T cell differentiation, and autoimmune pathogenesis [37]. PKC-θ overexpression has been demonstrated in gastrointestinal stromal tumors [38]; however, its main roles, including those in pancreatic cancer, have not been examined. Treatments with AH6809/GW627368X alone induced PKC-θ phosphorylation, indicating that EP2/EP4 signaling may negatively regulate PKC-θ phosphorylation. Moreover, the direct inhibition of PKC-θ using a pseudosubstrate caused further decrease in cell viability, as well as suppression of the phosphorylation of MEK and ERK. Thus, phosphorylated PKC-θ may be associated with cell survival, maintenance of cell viability, and cell proliferation. Inhibition of the basal activity of PKC-θ also suppressed IGF-1-mediated growth stimulation (data not shown). Some studies suggest that IGF-1/IGF-1R signaling directly upregulates the anti-apoptotic protein bcl-2 and stabilizes the integrin α5β1, which is associated with the MAPK pathway and cell growth, where its activation and/or function was influenced by PKC activity [39-42]. Based on these reports, we suggest that other cellular events may occur independently of EP2/EP4 signaling-mediated crosstalk in IGF-1-treated cells. Under these conditions, the impact of PKC-θ activity on cell survival (anti-apoptotic) and growth may increase. Thus, a treatment with AH6809/GW627368X activates PKC-θ, and the effect of the PKC-θ pseudosubstrate may further decrease the cell viability. PKC-θ knockdown impaired the effects of AH6809/GW627368X pretreatment and induced the expression of PKC-α mRNA, suggesting that the compensatory induction of PKC-α, following the knockdown of PKC-θ, protected against the effects of AH6809/GW627368X. This result suggests that an RNA interference-based therapy may be limited to molecular targets with compensatory isoforms, and thus specific inhibitors or pseudosubstrates with appropriate toxicity, *in vivo* stability, and pharmaceutical availability may be required. The phospho-antibody arrays also indicated that the phosphorylation of IRS-1, which is closely related to the activation of the phosphoinositide-3 kinase (PI3K)/Akt signaling pathway, was induced by AH6809/GW627368X pretreatment in IGF-1 treated cells. The PI3K/Akt pathway is a key molecule in another major IGF-1-mediated signaling pathway; however, we found no changes in of Akt phosphorylation, as described above. Bouzakri et al. reported that the phosphorylation of IRS-1 Ser636/639 is associated with the reduced phosphorylation of IRS-1 on tyrosine and subsequent reduction in PI3K/Akt activation [43]. This may explain why Akt phosphorylation remained unchanged under our experimental conditions.

PKC-θ was reported to be phosphorylated by the kinases PDK1, AMPK, and MAP4K3 [33]. Immunoblotting showed that there were no increases in PDK1 and AMPK phosphorylation or changes in MAP4K3 level with AH6809/GW627368X pretreatment. However, MAP4K3 knockdown prevented the suppression of growth stimulation, phosphorylation of PKC-θ, and downregulation of phospho-MEK and -ERK by AH6809/GW627368X pretreatment, whereas the negative control siRNA and PDK-1 knockdown did not. These data indicate that MAP4K3 is activated and that it phosphorylates PKC-θ in AH6809/GW627368X-pretreated IGF-1 treated cells. MAP4K3 (also known as GLK) activates JNK family proteins in response to cellular stress and is expressed in several tissues including pancreas [44]. MAP4K3 activity is also regulated by amino acid sufficiency and the relevant phosphorylation sites have been identified [45]. However, further investigations using currently unavailable anti-phospho-MAP4K3 antibodies or specific inhibitors of MAP4K3 are required to determine whether AH6809/GW627368X pretreatment directly activates MAP4K3. AH6809/GW627368X pretreatment may also produce cellular stress, including alterations in the amino acid content, so further studies of the relationship between EP2/EP4 signaling and cellular stress-induced signal transduction are required to fully characterize the role of MAP4K3 in the present conditions.

To confirm the interactions between EP2/EP4 and IGF-1R signaling *in vivo*, we established an orthotopic xenograft model where IGF-1R signaling stimulated tumor growth. Initially, we established a stable transfectant cell line expressing hmIGF-1, which grew more rapidly than the vector-control cells and responded to AH6809/GW627368X treatment. The apparent induction of tumor growth was observed after the injection of this transfectant into the pancreas of nude mice. Using this transfectant, we examined the effects of AH6809/GW627368X treatment, which suppressed tumor growth and decreased the number of Ki-67-positive cells. Moreover, immunohistochemical analyses revealed that IGF-1 expression was preserved during the experimental period, thereby indicating their similarity to the *in vitro* conditions that we utilized. Immunoblotting of tumor lesions also detected significant alterations in the phosphorylation status of PKC-θ and ERK, as shown *in vitro*. PGE_2_ also exerts various effects on the immune system. With respect to tumor immunity, PGE_2_ acts as an enhancer of tumor-suppressive regulatory T cells and macrophages (M2 macrophages). PGE_2_ also suppresses the function of NK cells and cytotoxic T lymphocytes, as well as contributes to the breakdown of the Th1/Th2 balance [46]. Thus, the mice in this model were athymic and treatment with AH6809/GW627368X could enhance the T cell-associated host tumor-immunity as well as obtaining additive and/or synergistic effects in general conditions, in addition to the effect of the EP antagonists itself. There was a strong agreement between the results of *in vitro* and *in vivo* experiments, so we performed clinico-pathological analyses of surgical specimens from pancreatic cancer patients. According to these analyses, 17.4% of the cases were quadruple-positive for IGF-1R, EP2/EP4, MAP4K3, and PKC-θ, indicating that crosstalk may occur between these signaling pathways in human pancreatic cancers. Report from Koshiba et al., which demonstrated that COX-2-positive rate in pancreatic cancer is very high [47], also assists the probability of this crosstalk. Although the quadruple-positive rate of 17.4% is not very high, this result suggests that our *in vitro* and *in vivo* findings may be reflected in the clinical stages. In addition, these molecules were detected by immunohistochemistry using formaldehyde-fixed paraffin blocks. Because the states of the paraffin blocks and the surgical specimens were highly variable, it is possible that this may have caused the positive rate for each parameter to appear relatively low. Therefore, the positive rate may increase if another detection method is applied (e.g., immunoblotting or RT-PCR using fresh samples). These results also suggest that the specific inhibition by PKC-θ may effectively suppress cancer cell viability via changes in EP2/EP4 signaling in cases that are quadruple-positive for IGF-1R, EP2 or EP4, MAP4K3, and PKC-θ. Thus, specific small-molecule inhibitors of PKC-θ could be developed for combination therapy with EP2/EP4 antagonists to exploit this novel molecular target for cancers that co-express IGF-1R, EP2 or EP4, and PKC-θ. Combinations of anti-IGF-1R antibodies or small molecule inhibitors of IGF-1R with gemcitabine, which are used widely to treat pancreatic cancer, have additive or synergistic effects on growth and survival [48, 49]. However, the presence of the EP2/EP4-MAP4K3-PKC-θ axis and the precise effects of gemcitabine on this remain unresolved.

Other unresolved mechanisms include: (i) the effects of EP2/EP4 signaling on HB-EGF-mediated cellular signaling; (ii) the involvement of other phospho-proteins identified in our phospho-antibody arrays; (iii) the relationships between EP2/EP4 antagonism and MAP4K3 activation; and (iv) the presence of these molecules in other types of cancer. Although the activities of these phospho-proteins have been reported in previous studies [50-52], our data indicate that multiple uncharacterized mechanisms require further study. Overall, our results demonstrate the crosstalk between EP2/EP4 and IGF-1R signaling mainly in pancreatic cancer cells that produce and secrete PGE_2_. The production of PGE_2_ is reportedly upregulated in numerous cancer types and the circulating IGF-1 levels are relatively stable. Therefore, these molecular interactions may occur in the presence of anti-inflammatory drugs or EP2/EP4 antagonists. In these cases, PKC-θ may be a potent therapeutic target for decreasing the cell growth and viability in tumor lesions.

## MATERIALS AND METHODS

### Cells, animals, and reagents

Human pancreatic cancer cell lines MiaPaCa-2, BxPC-3, PANC-1, and Capan-1 were purchased from the European Collection of Cell Cultures (Salisbury, UK). Cells were maintained in DMEM (MiaPaCa-2) and RPMI1640 (BxPC-3, PANC-1 and Capan-1) supplemented with 10% FBS, 100 unit/mL penicillin G and 0.1 mg/mL streptomycin sulfate. Five-week-old male nude mice were purchased from Charles River Japan (Yokohama, Japan), which were housed in specific pathogen-free conditions. The experimental protocols were performed in accordance with the Care and Use of Laboratory Animals of the University of Tokushima School of Medicine and were approved by the Animal Care and Use Committee. Recombinant human IGF-1 was purchased from PeproTech (Rocky Hill, NJ) and human HB-EGF and IGF-2 were purchased from R&D systems (Minneapolis, MN). The EP2-selective antagonist AH6809 and EP4-selective antagonist GW627368X were purchased from Cayman Chemical (Ann Arbor, MI). The pseudo-substrate of PKC-θ was purchased from Merck Millipore (Billerica, MA).

### Expression of mRNAs and assessment of PGE2 secretion using enzyme immunoassays

Total RNAs samples were isolated from MiaPaCa-2, BxPC-3, PANC-1, and Capan-1 cells using RNeasy Mini kits (QIAGEN, Valencia, CA). Aliquots (1 μg) were then subjected to semi-quantitative RT-PCR as previously described [53], and COX-1, COX-2, EP1, EP2, EP3, EP4, IR, IGF-1R, IGF-2R, EGFR, ErbB4, and NRDc were determined using β-actin mRNA as an internal control with the primer sequences listed in Supplementary Table 1. The PGE_2_ concentrations were determined in CM using a PGE_2_ Express EIA kit (Cayman Chemical).

### Growth stimulation assay

Cells (5 × 10^3^ cells/well or 3 × 10^5^ cells/well) were plated on 96-well microplates or six-well plates, preincubated overnight at 37°C, and then starved for 24 h in 100 μL or 1.5 mL of serum-free medium. Subsequently, equal volumes of serum-free medium containing HB-EGF, IGF-1, and IGF-2 (50, 20, and 50 ng/mL, respectively) were added, and the cells were incubated for 48 h or 20 min at 37°C. Pretreatments with 5 μM AH6809/GW627368X were performed for 3 h prior to the growth factor treatments. After stimulation, the viable cells were counted by the MTT method or subjected to immunoblotting. The immunoblotting procedure is described below.

### Immunoblotting

Untreated, treated, knockdown cells, and orthotopic tumor specimens were lysed and their protein levels were determined by immunoblotting, as previously described [53]. The antibodies used in this study are listed in Supplementary Table 2. Signals were detected using an Immobilon Western horseradish peroxidase substrate (Merck Millipore). Signals from tumor samples were quantified using ImageJ software and their relative intensities were calculated.

### Phospho-antibody array

IGF-1R signaling was assessed in IGF-1-stimulated and AH6809/GW627368X-pretreated IGF-1-stimulated BxPC-3 cells (5 × 10^6^) using a Phospho Antibody Array contract service (FullMoon BioSystems Inc; Filgen, Nagoya, Japan). The protein phosphorylation levels were normalized against the corresponding total protein concentrations and the fold changes in AH6809/GW627368X-pretreated IGF-1-stimulated BxPC-3 cells were expressed relative to those in IGF-1-stimulated BxPC-3 cells. The concentrations of IGF-1, AH6809, and GW627368X were the same as those used in the growth stimulation assays. Pretreatments and treatments were performed for 3 h and 20 min, respectively.

### RNA interference

BxPC-3 cells (3 × 10^5^ cells/well) were seeded onto six-well plates and preincubated overnight at 37°C. The following day, the cells were transfected with negative universal control siRNA (Life Technologies, Carlsbad, CA), PKC-θ siRNA (ID SASI_Hs01_00239143; Sigma), MAP4K3 siRNA (ID SASI_Hs02_00335960 and SASI_Hs01_00040140; Sigma) and PDK1 siRNA (ID SASI_Hs01_00094378 and SASI_Hs01_00044485; Sigma) using lipofectamine RNAiMAX (Life Technologies), according to the manufacturer's protocol. After transfection, we analyzed the effects of AH6809/GW627368X on IGF-1-stimulated cell growth and the activations of PKC-θ, MEK, and ERK with a growth stimulation assay and immunoblotting using the conditions and procedures described above. Total RNA samples were isolated from negative control siRNA- and PKC-θ siRNA-transfected cells using RNeasy Mini kits, and aliquots (1 μg/sample) were then subjected to semi-quantitative RT-PCR. The conditions and procedures are previously described [53]. The levels of PKC-α, PKC-β, PKC-γ, PKC-δ, PKC-ε, PKC-η, PKC-θ, PKC-ζ, and PKC-ι mRNA were quantified using the primer sequences listed in Supplementary Table 1. β-actin mRNA was used as an internal standard.

### Orthotopic xenograft model in nude mice

Mice were anesthetized with ketamine and xylazine (1.8 and 0.8 mg/mouse, respectively) and surgery was performed. Briefly, a small left flank incision was made and the pancreas was exteriorized. Intrapancreatic injections of BxPC-hmIGF1 cells (1.2 × 10^6^ cells/mouse) were then performed using a 30-gauge syringe. To avoid intraperitoneal leakage of the cell suspensions, the injected region was pressed with a sterilized cotton swab for 10 s. Both layers of the abdominal wounds were closed with wound clips. The mice were then treated daily with intraperitoneal saline (n = 10) or 4 mg/kg AH6809/GW627368X (n = 10) from the next day. After 35 days, the mice were euthanized and their tumor lesions were collected and weighed. Parts of the tumor specimens were retained for immunoblotting and the remaining tumor tissues were then fixed in 10% phosphate-buffered formaldehyde.

### Histological analyses

Formaldehyde-fixed tissues were embedded in paraffin and sectioned at 4 μm. All of the sections were subjected to H&E staining and immunohistochemical staining for IGF-1 and Ki-67. Quantitative analyses were performed by microscopically counting the numbers of Ki-67-positive cells per field. The antibodies used in this study are listed in Supplementary Table 2. The sections were blocked with 1% hydrogen peroxide in 50% methanol before probing with primary antibodies. The antigens on the paraffin sections were then retrieved by autoclaving in 0.01 M citrate buffer (pH 6.0) for 10 min and visualized using a ChemMate Envision kit with a horseradish peroxidase/3,3′-diaminobenzidine kit. All sections were counterstained with Mayer's hematoxylin (Muto Pure Chemicals, Tokyo, Japan).

### Clinico-pathological analysis

Formaldehyde-fixed paraffin blocks from 23 surgically resected pancreatic cancers were obtained from Tokushima University Hospital (2005-2011). Serial sections (4 μm thickness) were cut and immunohistochemical staining was performed for IGF1R, EP2, EP4, MAP4K3, and PKC-θ. Pathologists differentiated the slides as positive or negative for antigens in a double-blinded test. The antibodies used in this study are listed in Supplementary Table 2. Antigen retrieval, visualization, and counterstaining were performed as described above.

### Statistical analysis

The body weights, tumor weights, Ki-67-labeling indices, and immunoblotting data were compared using two-tailed Mann-Whitney U tests. All other comparisons were performed using two-tailed Student's *t*-tests and the differences were considered statistically significant when *P* < 0.05.

## SUPPLEMENTARY MATERIAL FIGURES AND TABLES


