# Supplementary material for: Inhibition of EP2/EP4 signaling abrogates IGF-1R-mediated cancer cell growth: Involvement of protein kinase C-θ activation
Source: Oncotarget. 2014 Dec 31;6(7):4829–44. doi: 10.18632/oncotarget.3104 (PMC4467118; doi:10.18632/oncotarget.3104)
Supplement: Supplementary file 1 [file oncotarget-06-4829-s001.pdf]

Inhibition of EP2/EP4 signaling abrogates IGF-1R-mediated cancer cell growth: Involvement of protein kinase C-θ activation

Supplementary Material

Supplementary Table 1: Primer sequences for semi-quantitative RT-PCR.

Target	Forward (5’→3’)	Reverse (5’→3’)
COX-1	GCCGGAGTCTCTTGCTCCGG	GGGCTGGGCCGCAGTGAATT
COX-2	CGCAGTACAGAAAGTATC	CTCTGGATCTGGAACAC
EP1	CCATGGTTATGAGCCCTTGCGGGC	AGATCTTTAGAAGTGGCTGAGGC
EP2	GAATTCGATGGGCAATGCCTCCAATG	AGATCTAAGGTCAGCCTGTTTACTGGC
EP3	GAATTCGATGAAGGAGACCCGGGGCTAC	GAATTCCTACTTGGGAGGCTGAG
EP4	CCATGGTTATGTCCACTCCCGGGGTC	AGATCTTTATATACATTTTTCTG
IR	AGGGCTCCTTCGGCATGG	CCCAGGAGGCGCACCACA
IGF-1R	CCTGGGCCAGTGACCTGGGA	TTCACGCATGCTTGCGGCCT
IGF-2R	GCACCTTTGCAGTCGGGCCA	CACAGGGGACGCCGTCATCG
EGFR	AGCTGAAGACGTTATCATCTGTGCC	GGCAGACCAGGCAGTCGCTC
ErbB4	CGCCCTTCAGCACGGGATCTG	GCCGCCACGAGAAGGCTCAC
NRDc	CGCATAACCTTGCGGAACCAGC	GGCCAAAGTCTCAGGCTTGATGAGG
PKC-α	CTTCACACGAGGACAGCC	CTTGTTCAGCAACGTCCT
PKC-β	ACCGCCTGTACTTTGTGA	CCACAATAGCCGTTGAGC
PKC-γ	GACCCCCGGAACAAACAC	TTGTACCAGCCATCCACG
PKC-δ	GCCGCTTTGAACTCTACC	CCAACCTCCGCTTTTCCA
PKC-ε	AAAATCACCAACAGCGGC	ATGCTGGTGGAGGAACAT
PKC-η	GGCTCACCGGACGGGTA	GAAACTCCCGGTAAGGGT

PKC-θ	CTGTGGACTGGTGGTCCTTC	CCGTTTGCCCCTGATTCAAC
PKC-ζ	ACTGCAAACTGCTGGTCC	ACCGACTTGTCGTCTGGA
PKC-ι	GTTGGGCTGCATTCTTGC	GTCATCTGGAGTGAGCTG
β-actin	TACAATGAGCTGCGTGTGG	AGATGGGCACAGTGTGGG

Supplementary Table 2: Antibodies used in this study

Immunogen	Source	Application	Manufacturer	Clone No.	Dilution
**Primary**					
pERK1/2 (Thr202/204)	Rabbit mono	IB*	Cell Signaling	197G2	1:1,000
ERK1/2	Rabbit mono	IB	Cell Signaling	137F5	1:1,000
pMEK1/2 (Ser221)	Rabbit mono	IB	Cell Signaling	166F8	1:1,000
MEK1/2	Rabbit mono	IB	Cell Signaling	47E6	1:1,000
pPKC-θ (Thr538)	Rabbit poly	IB	Cell Signaling	-	1:500
PKC-θ	Mouse mono	IB	BD Transduciton	27	1:500
pPDK1 (Ser241)	Rabbit mono	IB	Cell Signaling	S241	1:1,000
PDK1	Rabbit poly	IB	Cell Signaling		1:1,000
pAMPKα (Thr172)	Rabbit mono	IB	Cell Signaling	40H9	1:1,000
AMPKα	Rabbit poly	IB	Cell Signaling		1:1,000
MAP4K3	Rabbit poly	IB	Cell Signaling		1:1,000
Actin	Rabbit poly	IB	Sigma		1:10,000
Ki-67	Mouse mono	IHC*	Dako	MIB-1	1:100
IGF-1	Rabbit poly	IHC	Abcam		1:100

IGF-1R	Rabbit poly	IHC, IB	Abcam		1:500 (WB)<br>1:50 (IHC)
EP2	Rabbit mono	IHC	Abcam	EPR8030	1:4,000
EP4	Rabbit poly	IHC, IB	Abcam		1:1,000 (WB)<br>1:100 (IHC)
PKC-θ	Rabbit poly	IHC	Abcam		1:50
MAP4K3	Rabbit poly	IHC	Abcam		1:200
**Secondary**					
Rabbit IgG (H+L)	Goat poly	IB	Jackson		1:100,000
Mouse IgG (H+L)	Goat poly	IB	Thermo		1:50,000

*Abbreviations: IB, immunoblotting, IHC, immunohistochemical staining.

Supplementary Table 3: Primer sequences for pIRES-hmIGF1 construction

Name	Purpose	Sequence (5’→3’)
GGC-Eco-G-hmIGF1-F*	To make the PCR fragment for subcloning to pFUSE-hFc2 (IL2ss) vector	GGCGAATTCGGGGACCGGAGACGCTCTGC
GGC-Bam-hmIGF1-R*		GGCGGATCCAGCTGACTTGGCAGGCTT
GGC-Age-IL2ss-F**	To make the PCR fragment for subcloning to pIRESneo3 vector	GGCACCGGTGCCGCCACCATGTACAGGATGCAA
GGC-Not-hmIGF1-R**		GGCGCGGCCGCTCAAGCTGACTTGGCAGG

*Subcloned vector by this primer set was designated as pFUSE-hmIGF1/Fc.

**PCR by this primer set was performed using pFUSE-hmIGF1/Fc as a template.

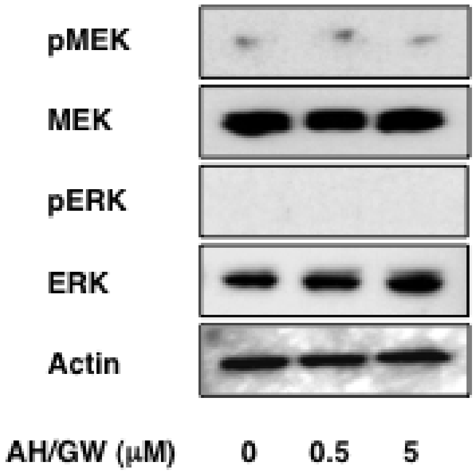

Supplementary Figure 1: Effects of the EP2/EP4 antagonists AH6809/GW627368X on MEK and ERK activation in BxPC-3 cells. BxPC-3 cells were treated with 0.5 and 5 μM AH6809/GW627368X for 3 h and the levels of phosphorylated MEK and ERK were determined by immunoblotting.

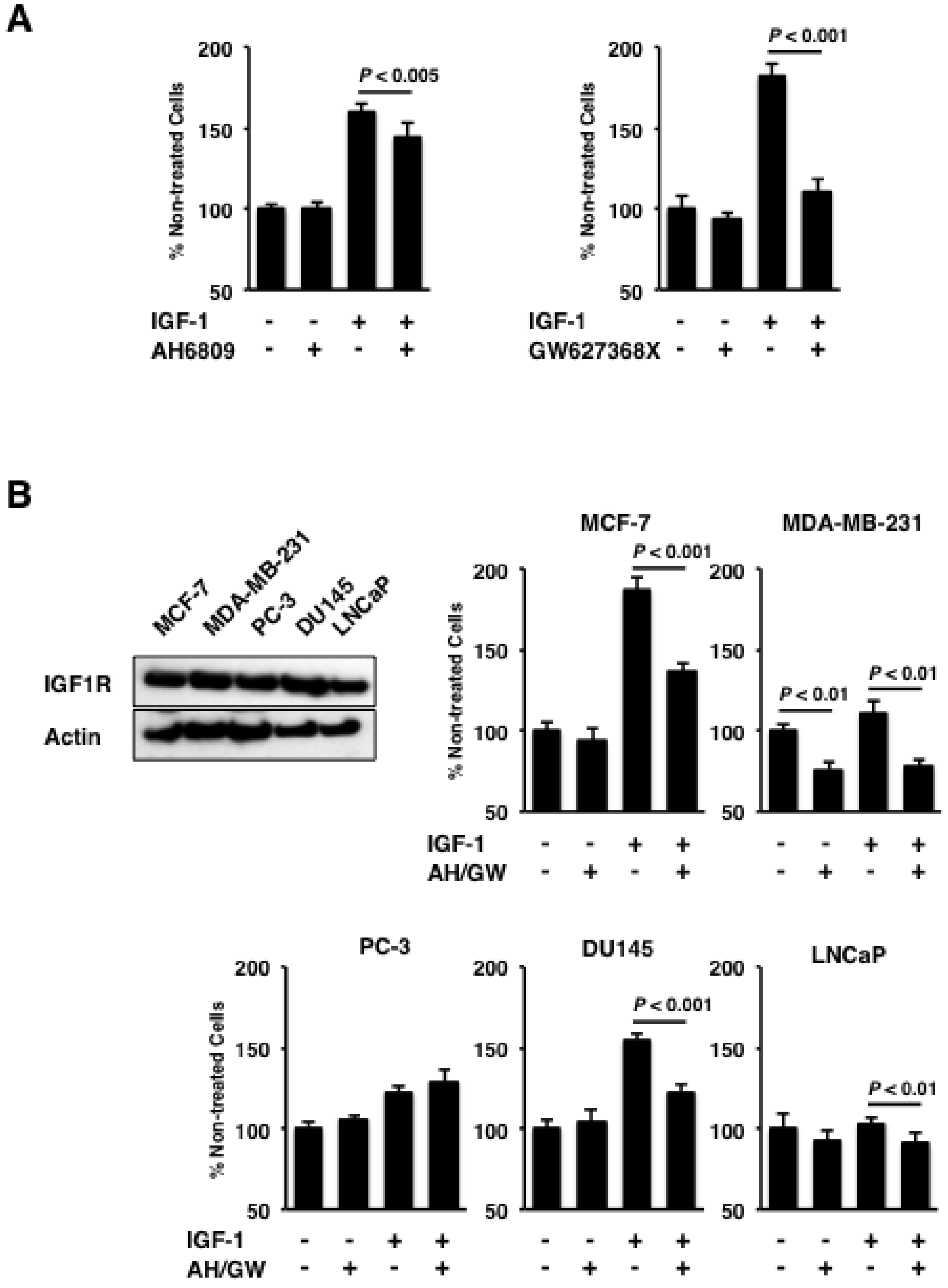

Supplementary Figure 2: Single effects of AH6809 and GW627368X in BxPC-3 cells, and the effect of AH6809/GW627368X on IGF-1-stimulated growth in prostate and breast cancer cell lines. A, BxPC-3 cells were stimulated with or without IGF-1 (20

ng/mL) for 48 h in the absence or presence of AH6809 or GW627368X (5 μM) pretreatment for 3 h, and cell growth was measured by the MTT assay. The A_{550} values for untreated cells were assigned as 100% and the relative percentages for treated cells are shown. *Columns*, mean percentages (n = 6); *bars*, SD. B, Expression of IGF-1R protein in PC-3, DU145, LNCaP, MDA-MB-231, and MCF-7 cells was confirmed by immunoblotting. Next, these cells were stimulated with or without IGF-1 (20 ng/mL) for 48 h in the absence or presence of AH6809/GW627368X (5 μM each) pretreatment for 3 h, and cell growth was measured by the MTT assay. The A_{550} values for untreated cells were assigned as 100% and the relative percentages for treated cells are shown. *Columns*, mean percentages (n = 6); *bars*, SD.

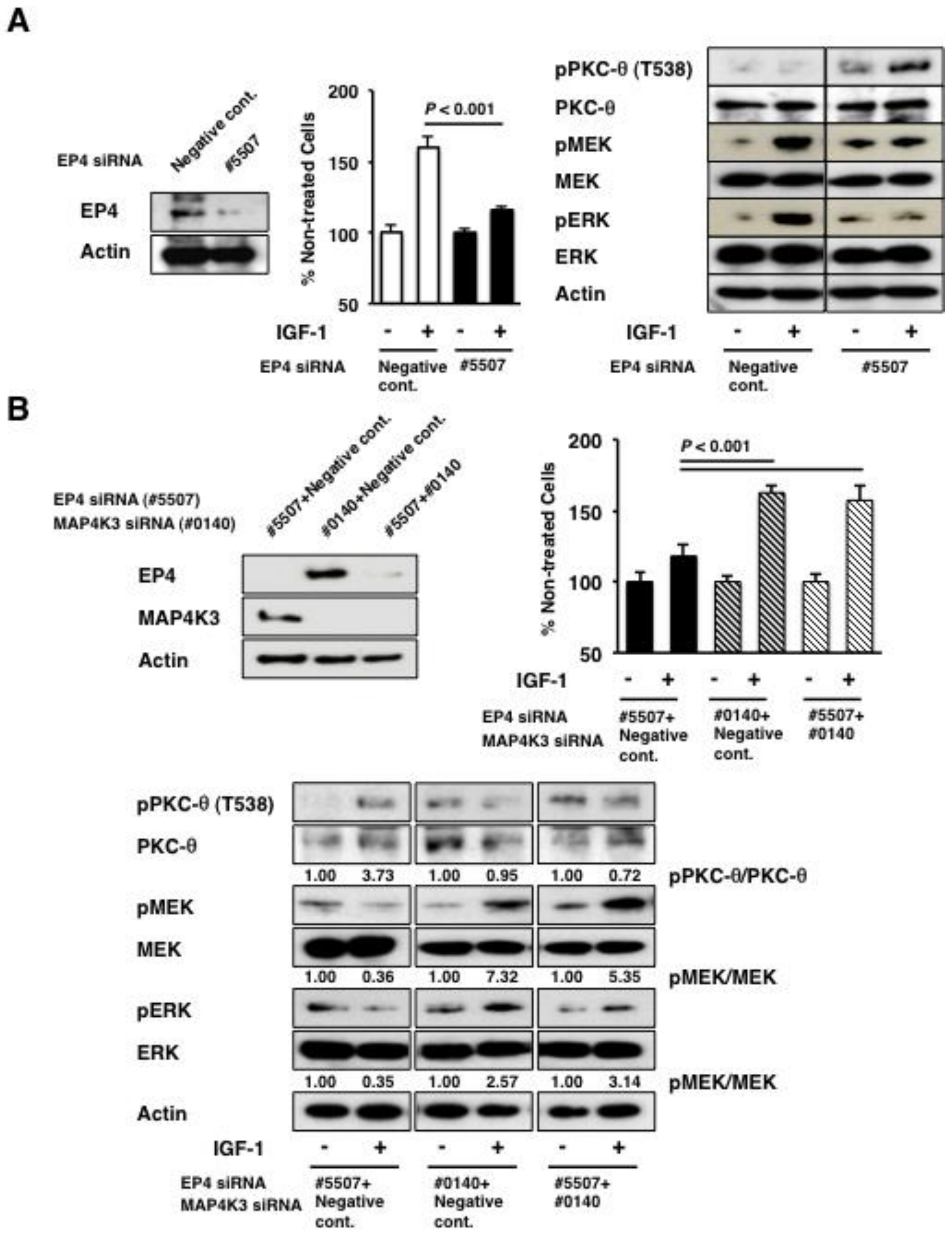

Supplementary Figure 3: EP4 knockdown had the same effectiveness as treatment with EP2/EP4 antagonists. A, Knockdown using a specific siRNA for EP4 was performed in BxPC-3 cells, which was confirmed by immunoblotting. Cells transfected

with negative control siRNA and EP4 siRNA were stimulated with IGF-1 for 48 h or 20 min and then tested in growth stimulation assays and immunoblotting. The A_{550} values for untreated cells were assigned as 100% and the relative percentages for treated cells are shown. *Columns*, mean percentages (n = 6); *bars*, SD. B, Double-knockdown using specific siRNAs for EP4 and MAP4K3 was performed in BxPC-3 cells. Cells were transfected with negative control siRNA plus EP4 siRNA, negative control siRNA plus MAP4K3 siRNA, or EP4 siRNA plus MAP4K3 siRNA. Knockdown was confirmed by immunoblotting. Transfected cells were stimulated with IGF-1 for 48 h or 20 min and tested using growth stimulation assays and immunoblotting. The A_{550} values for untreated cells were assigned as 100% and the relative percentages for treated cells are shown. The relative levels of phospho-PKC-θ, -MEK, and -ERK were calculated using ImageJ software. *Columns*, mean percentages (n = 6); *bars*, SD.

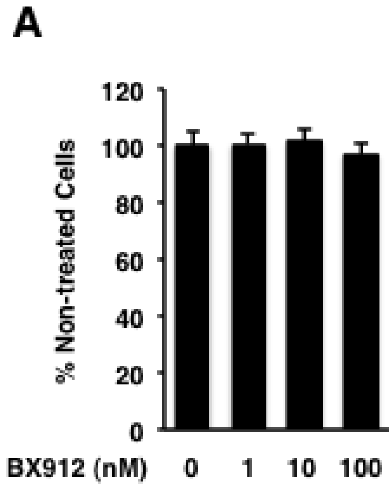

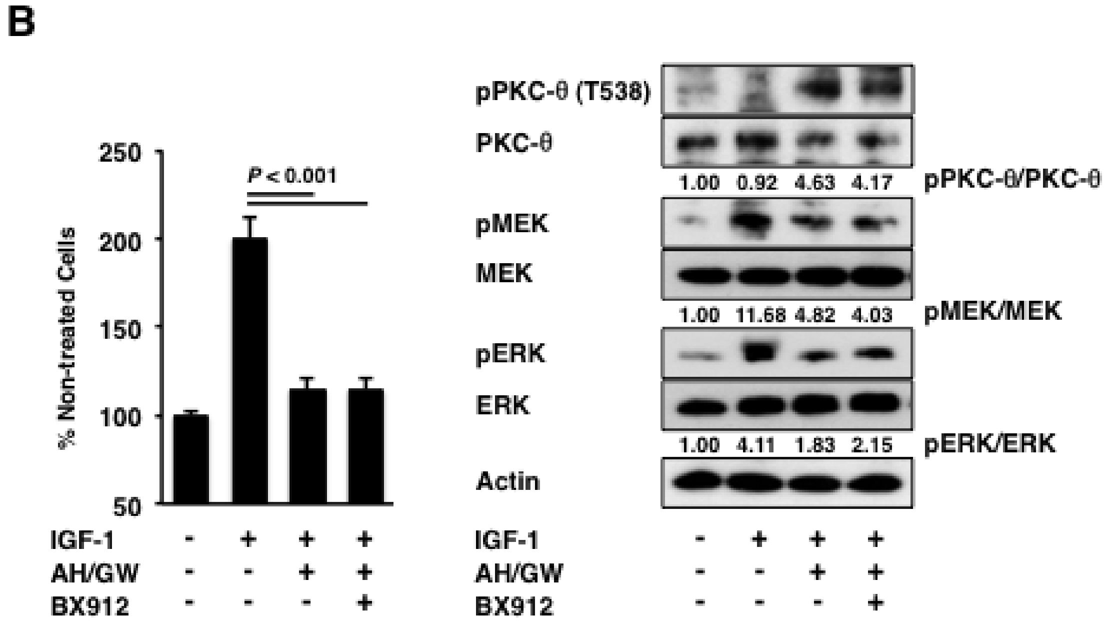

Supplementary Figure 4: The specific PDK1 inhibitor BX912 did not affect the activation of PKC-θ and subsequent cellular responses to EP2/EP4 antagonism. A, BxPC-3 cells were treated with 1, 10, and 100 μM BX912 for 48 h in serum-free medium and the cell viability was assessed using MTT assays. The A_{550} values for untreated cells were assigned as 100% and the relative percentages for treated cells are shown. *Columns*, mean percentages (n = 6); *bars*, SD. B, BxPC-3 cells were stimulated with IGF-1 for 20 min in the absence or presence of AH6809/GW627368X and BX912

(100 nM) pretreatment for 3 h. Cell growth and phosphorylation of PKC-θ, MEK, and ERK were determined by MTT assays and immunoblotting. The A_{550} values for untreated cells were assigned as 100% and the relative percentages for treated cells are shown. The relative levels of phospho-PKC-θ, -MEK and -ERK were calculated using ImageJ software. *Columns*, mean percentages (n = 6); *bars*, SD.

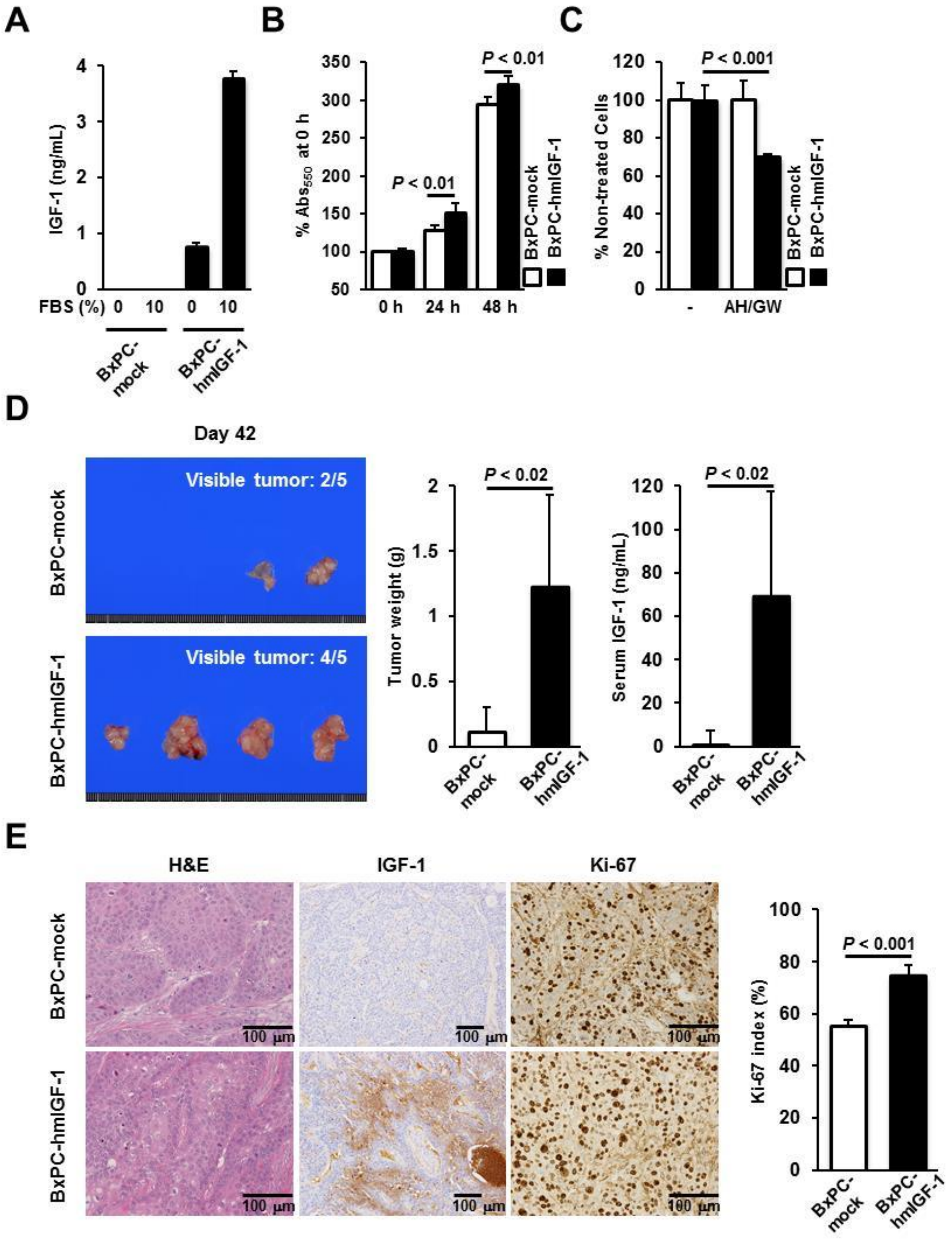
A
IGF-1 (ng/mL)
FBS (%) 0 10 0 10
BxPC-mock
BxPC-hmIGF-1
B
% Abs550 at 0 h
P < 0.01
P < 0.01
0 h 24 h 48 h
BxPC-mock
BxPC-hmIGF-1
C
% Non-treated Cells
P < 0.001
- AH/GW
BxPC-mock
BxPC-hmIGF-1
D
Day 42
BxPC-mock
Visible tumor: 2/5
BxPC-hmIGF-1
Visible tumor: 4/5
Tumor weight (g)
P < 0.02
BxPC-mock
BxPC-hmIGF-1
Serum IGF-1 (ng/mL)
P < 0.02
BxPC-mock
BxPC-hmIGF-1
E
H&E
IGF-1
Ki-67
BxPC-mock
BxPC-hmIGF-1
100 μm
Ki-67 index (%)
P < 0.001
BxPC-mock
BxPC-hmIGF-1

Supplementary Figure 5; Expression of hmIGF-1 accelerates *in vitro* and *in vivo* pancreatic cancer cell growth. A, The secretion of hIGF-1 in serum-free medium or complete culture medium were measured by ELISA. *Columns*, mean (n = 4); *bars*, SD. B, BxPC-mock and BxPC-hmIGF1 cells were incubated in complete medium for 24 and 48 h, and cell proliferation was measured using the MTT assay. The A_{550} value at 0 h was assigned as 100% and the relative percentages at 24 and 48 h are shown. *Columns*, mean percentages (n = 6); *bars*, SD. C, BxPC-mock and BxPC-hmIGF1 were incubated with 5 μM AH6809/GW627368X for 48 h in serum-free medium, and the cell viability was measured using MTT assays. The A_{550} values for untreated cells were assigned as 100% and the relative percentages for treated cells are shown. *Columns*, mean percentages (n = 6); *bars*, SD. D, Image of a tumor lesion from a BxPC-mock- and BxPC-hmIGF1-injected mouse. Average tumor weights and serum hIGF-1 levels were measured. *Columns*, mean; *bars*, SD. E, H&E staining and immunohistochemical staining of IGF-1 and Ki-67 in tumor lesions. The percentages of Ki-67-positive cells were calculated. *Columns*, mean; *bars*, SD.

Supplementary Materials and Methods

Effect of AH6809/GW627368X on the activation of MEK and ERK in BxPC-3 cells

BxPC-3 cells (3×10^5 cells/well) were plated onto six-well plates, preincubated overnight at 37°C, and then starved for 24 h in 1.5 mL of serum-free medium. Subsequently, the cells were treated with AH6809 and GW627368X (0.5 and 5 μM, respectively) for 3 h at 37°C. After treatment, the cells were subjected to immunoblotting for phospho-MEK, MEK, phospho-ERK, ERK, and actin.

EP4 knockdown studies

BxPC-3 cells (3×10^5 cells/well) were seeded onto six-well plates and preincubated overnight at 37°C. The following day, the cells were transfected with negative universal control siRNA and EP4 siRNA (ID SASI_Hs01_00105507; Sigma) using Lipofectamine RNAiMAX, according to the manufacturer's protocol. After transfection, the cells (5×10^3 or 3×10^5 cells/well) were re-plated onto 96-well microplates or six-well plates. After preincubation overnight, the cells were starved for 24 h in serum-free medium and then stimulated with IGF-1 for 48 h or 20 min. Cell growth and the phosphorylation of MEK and ERK were determined by MTT assays and immunoblotting, respectively.

Effect of BX912 on the AH6809/GW627368X-mediated abrogation of IGF-1-induced cellular growth and signaling

The effect of BX912 on cell viability was determined by MTT assays. BxPC-3 cells (5

× 10^3 cells/well) were plated onto 96-well microplates, preincubated overnight at 37°C, and then treated with 1, 10, or 100 nM BX912 (SYNkinase, San Diego, CA) for 48 h in serum-free medium at 37°C. After treatment, the viable cells were counted using the MTT method. The effect of BX912 on the AH6809/GW627368X-mediated abrogation of IGF-1-induced cellular growth and signaling was determined based on growth stimulation assays and immunoblotting. Briefly, BxPC-3 cells (5 × 10^3 or 3 × 10^5 cells/well) were plated onto 96-well microplates or six-well plates. After preincubation overnight, the cells were starved for 24 h in serum-free medium and then stimulated with IGF-1 for 48 h or 20 min in the absence or presence of AH6809/GW627368X and BX912 (100 nM) pretreatment for 3 h. Cell growth and the phosphorylation of PKC-θ, MEK, and ERK were then determined by MTT assays and immunoblotting, respectively.

Establishment of stable transfectants expressing hmIGF-1

Total RNA was isolated from human Ewing sarcoma SK-ES-1 cells and cDNA that contained the complete coding sequence of human mature IGF-1 (hmIGF-1) was synthesized and amplified by PCR, as previously described [47]. The PCR products were digested with *Eco*RI and *Bam*HI, and then subcloned into pFUSE-hFc2 (IL2ss) vectors (Invivogen, San Diego, CA). Secondary PCR was then performed using this construct as a template. The PCR products were then digested with *Age*I and *Not*I, and the products were subcloned into pIRESneo3 vectors (Clontech, Mountain View, CA). The construct obtained was designated as pIRES-hmIGF1. Successful constructs were confirmed by direct sequencing with an ABI PRISM 3130xl Genetic Analyzer (Life Technologies). The primer sequences are listed in Supplementary Table 3. BxPC-3 cells were transfected with pIRESneo3 or pIRES-hmIGF1 using Lipofectamine LTX (Life

Technologies), according to the manufacturer's protocol. Transfected cells were treated with 1 mg/mL G418 sulfate (Promega, Madison, WI) every 3 days for 2 weeks. After selection, the stable transfectants were maintained in complete CM containing 0.1 mg/mL G418 sulfate and designated as BxPC-mock and BxPC-hmIGF1 cells, respectively. The BxPC-hmIGF1 cells were examined using hIGF-1 ELISA, cell proliferation assays, tests of proliferative reactions after treatment with AH6809/GW627368X, and *in vivo* growth assays in an orthotopic xenograft model. These methods are described below.

***In vitro* characteristics of BxPC-hmIGF1**

hIGF ELISA was performed in CM from BxPC-mock and BxPC-hmIGF1 cells (4×10^5/2 mL/72 h in complete or serum-free CM), and the production of hIGF-1 was quantified using a Quantikine human IGF-1 ELISA kit (R&D Systems, Minneapolis, MN). Cell proliferation assays with BxPC-mock and BxPC-hmIGF1 cells (5×10^3 cells/well) were performed after seeding the cells onto 96-well microplates and preincubating overnight at 37°C. Subsequently, CM was replaced with 100 μL fresh complete medium. After incubation for 24, 48, and 72 h, the viable cells were counted using the MTT method. Tests of the proliferative responses to treatments with AH6809/GW627368X were performed in BxPC-mock and BxPC-hmIGF1 cells (5×10^3 cells/well) after seeding onto 96-well microplates and preincubating overnight at 37°C. CM was then replaced with 100 μL of serum-free medium and cells were further incubated overnight at 37°C. Finally, the cells were treated with 5 □M AH6809/GW627368X and incubated for 48 h before counting the viable cells using the MTT method.

***In vivo* characteristics of BxPC-hmIGF-1**

In vivo growth assays were performed in an orthotopic nude mouse xenograft model. The mice were anesthetized with ketamine/xylazine and BxPC-mock or BxPC-hmIGF1 cells (1×10^6 cells/mouse) were injected intrapancreatically (n = 5 in the BxPC-mock group and n = 5 in the BxPC-hmIGF1 group). After 42 days of observation, the mice were euthanized and the tumor lesions were collected and weighed. The tumors were then fixed in 10% phosphate-buffered formaldehyde for the histological analyses. Whole blood was also collected from all the mice and sera were obtained. hIGF ELISA was performed to assess the persistence of hmIGF-1 expression. Formaldehyde-fixed tissues were embedded in paraffin and sectioned at 4 μm. All of the sections were subjected to H&E staining and immunohistochemical staining for IGF-1 and Ki-67. Quantitative analyses were microscopically performed by counting the number of Ki-67-positive cells per field.
